# Possibility of independent use of the yes/no Angoff and Hofstee methods for the standard setting of the Korean Medical Licensing Examination written test: a descriptive study

**DOI:** 10.3352/jeehp.2022.19.33

**Published:** 2022-12-12

**Authors:** Do-Hwan Kim, Ye Ji Kang, Hoon-Ki Park

**Affiliations:** 1 Department of Medical Education, Hanyang University College of Medicine, Seoul, Korea; 2 Department of Family Medicine, Hanyang University College of Medicine, Seoul, Korea; Hallym University, Korea

**Keywords:** Educational measurement, Humans, Physicians, Republic of Korea, Undergraduate medical education

## Abstract

**Purpose:**

This study aims to apply the yes/no Angoff and Hofstee methods to actual Korean Medical Licensing Examination (KMLE) 2022 written examination data to estimate cut scores for the written KMLE.

**Methods:**

Fourteen panelists gathered to derive the cut score of the 86th KMLE written examination data using the yes/no Angoff method. The panel reviewed the items individually before the meeting and shared their respective understanding of the minimum-competency physician. The standard setting process was conducted in 5 rounds over a total of 800 minutes. In addition, 2 rounds of the Hofstee method were conducted before starting the standard setting process and after the second round of yes/no Angoff.

**Results:**

For yes/no Angoff, as each round progressed, the panel’s opinion gradually converged to a cut score of 198 points, and the final passing rate was 95.1%. The Hofstee cut score was 208 points out of a maximum 320 with a passing rate of 92.1% at the first round. It scored 204 points with a passing rate of 93.3% in the second round.

**Conclusion:**

The difference between the cut scores obtained through yes/no Angoff and Hofstee methods did not exceed 2% points, and they were within the range of cut scores from previous studies. In both methods, the difference between the panelists decreased as rounds were repeated. Overall, our findings suggest the acceptability of cut scores and the possibility of independent use of both methods.

## Introduction

### Background/rationale

The Korean Medical Licensing Examination (KMLE) is a test that evaluates whether a physician has the appropriate ability to perform professional activities. The KMLE measures whether “individual medical graduates” can be accredited as professionals. It is critical to establish the cut score that determines whether the candidate passes the KMLE. The KMLE began in 1952, and discussions on cut scores have been underway since the early 2000s [1]. Examinees must get equal to or more than 60% of the total score and equal to or more than 40% in each subject to pass the written test of KMLE [2]. These standards have been used for a long time because they are specified in regulations. However, they are used conventionally without considering why the cut-off score was established this way, and it is criticized for lack of measurement grounds [3]. The score of 60% or more of the total score is generally a very familiar number when considering passing criteria. It has been used as a universal criterion for indicating the minimum competency for a long time. However, there is also an opinion that it is unreasonable to accept these figures as a standard for determining whether qualified health professionals have adequate abilities [4].

Given the increasing importance of the appropriate standard setting for high-stake exams, attempts have been reported to compare different standard setting approaches in licensing exams for health professionals. For example, in undergraduate nursing education, Yim and Shin [5] reported the findings derived from applying the Angoff method to a mock exam for the Korean Nursing Licensing Examination. According to their results, the final cut score was about 75% of the total score, which is about 15% points higher than the conventional 60%. In graduate medical education, Bourque et al. [6] compared the Ebel scores of the Royal College of Physicians and Surgeons of Canada certification exam by changing several conditions. They concluded that the specialties of the panelists and the availability of correct answers did not affect the final cut score [6]. Recently, in medical education, a simulation study examined the possibility of using the yes/no Angoff instead of the percent Angoff method for KMLE. Although the authors concluded the results of the yes/no Angoff are less reliable, the study was limited by its use of hypothetical yes/no Angoff data converted from percent Angoff data [7].

### Objectives

The current passing score of the KMLE written examination has a limitation because it has an arbitrary number of 60% as a conventional fixed cut score. To improve this practice, this study, aimed to apply the yes/no Angoff method to actual KMLE 2022 written examination data. At the same time, we also used 2 rounds of the Hofstee method to determine the cut score and compared the cut scores estimated from each method.

## Methods

### Ethics statement

Since this study was a secondary analysis using de-identified data provided by the Korea Health Personnel Licensing Examination Institute, neither approval from the Institutional Review Board nor the obtainment of informed consent is required.

### Study design

This exploratory descriptive study compares cut scores obtained from 2 standard-setting processes (the yes/no Angoff and Hofstee methods) to a conventional cut score (i.e., 60% of the total score). Specifically, among the variations of the Angoff method, we adopted the yes/no Angoff method that requires judges to make dichotomous categorical decisions. The yes/no Angoff method has an advantage because it places less cognitive burden than estimating probabilities [7].

### Setting

Unlike previous KMLEs, which were conducted in paper-based format, the 86th KMLE 2022, which was held from January 6 to 7, 2022, was the first exam that introduced computer-based testing in the written examination part [8]. A total of 320 items were presented. A total of 3,305 examinees took the computer-based testing for a written exam, and the overall passing rate was 96.6%. On January 14th and 15th, 2022, a panel meeting was held to derive a cut score for the 86th KMLE written test result data.

### Participants

It is essential to select a panel with content expertise in setting the standards. There is some difference in the appropriate number of panelists depending on the literature from at least 4–6 people to as many as 20 [9,10]. In this study, 14 panelists were selected based on their specialties and previous experience in developing and reviewing KMLE items.

### Study outcomes

The primary outcome of this study is the cut score when using the yes/no Angoff method and Hofstee method. As the secondary outcome, the passing rates were calculated based on the cut score obtained from both methods.

### Data sources/measurement

Before the panel meeting, the panelists must review all 320 KMLE items. They rated the importance and frequency of each item using a 3-point scale. In addition, through discussions on the characteristics of a minimum-competency physician, the panelists’ opinion on the minimum level of knowledge, skill, and ability required to perform the tasks of a physician was shared before starting the first round of voting for the standard setting process.

Details of the standard setting process using the yes/no Angoff are presented in Table 1. The work was done over 5 rounds. The first round generally corresponds to determining whether the borderline examinee can answer each item based on the content and difficulty. While looking at each item from the examinee’s perspective, each panelist estimated and submitted the possibility of correct answers of the minimum ability group as 0 or 1. Of the 5 rounds, the most time is devoted to the first round. In this study, 450 minutes were devoted, including the discussion time.

In the second round, the previous round’s results were shown before voting. The probability of correct answers for each item and cut scores were obtained by adding all estimated correct answers presented by each panelist. Based on these results, the panel can see how their opinions differ. The average value of the cut score estimated by each panelist was obtained, and the total cut score and estimated passing rates were shown. Finally, at the end of the fifth round, there was no more meaningful change in the cut score and acceptance rate, so the process was terminated.

The Hofstee method requires the panelists to submit 4 values: minimum and maximum values of acceptable passing scores (C_min and C_max) and minimum and maximum values of acceptable failure rates (F_min and F_max). A cumulative frequency distribution graph is drawn based on the actual scores of examinees, and the point where the straight line connecting (C_min, F_max) and (C_max, F_min) meet this graph is selected as the passing score. In this study, the Hofstee method was conducted twice: before starting the standard setting process and after the second round of the yes/no Angoff.

### Bias

The concept of the borderline examinee (i.e., minimal competence) may differ among the panelists due to their understanding of primary medical care. To supplement this, panelists who have experience as KMLE item developers provided a briefing on the background of the items to help other panelists fully understand the contents and goal of items.

### Study size

Since this study was not intended to test the effectiveness of a specific intervention, the sample size was not calculated, and the performance data of all examinees for the written test of the KMLE were analyzed.

### Statistical methods

In the standard setting process using the yes/no Angoff method, a cut score was derived by calculating the mean value of the votes collected from the panel using descriptive statistics.

## Results

### Participants

The panel consisted of 14 people (Table 2). There were 4 internal medicine specialists and family medicine specialists. Six panelists were from the departments of surgery, obstetrics and gynecology, pediatrics, psychiatry, preventive medicine, and emergency medicine. Two had experience in KMLE item development, and 5 had experience in KMLE item review.

### Main results

**Fig. 1.**
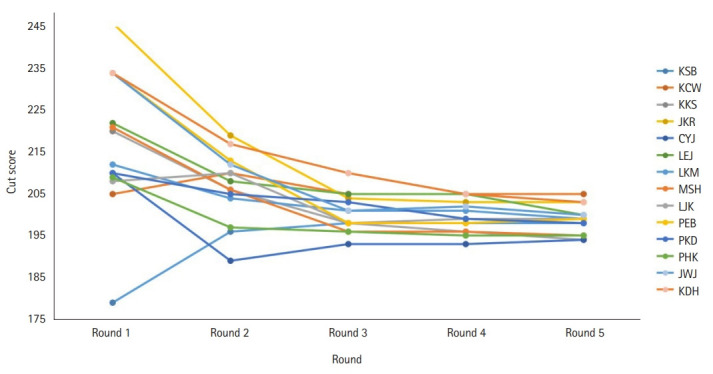
Change of cut scores of 14 panelists that converged toward a specific cut score during the yes/no Angoff method for the standard setting of 2022 Korean Medical Licensing Examination written test.

**Fig. 2.**
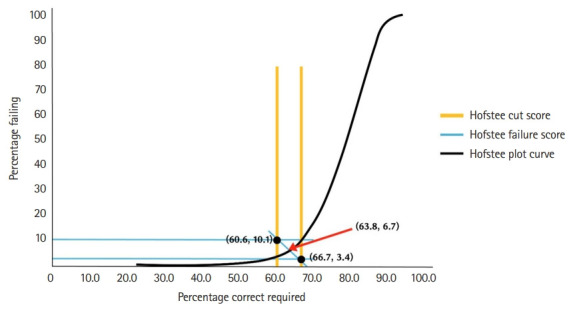
Derivation of cut score and failure rate of 2022 Korean Medical Licensing Examination written test by the Hofstee method. The x-axis is the percentage correct scores and y-axis is the failure rate.

For the yes/no Angoff method, as the round progressed, the standard error of the panel opinion gradually decreased and converged toward a specific cut score (Fig. 1, Table 3), from 67.8% (217 points, first round) to 61.9% (198 points, fifth round) based on 320 questions. Compared to the first and second rounds, the cut score and passing rate change became minimal in the fourth and fifth rounds. For the Hofstee method, before the standard setting process, the acceptable failure rates submitted by the panelists were 3.43% (minimum) and 11.3% (maximum), and the cut score was 61.4% (minimum) and 70.4% (maximum). Accordingly, the cut score was 65.0% (208 points) and the failure rate was 7.92%. After the second round, the acceptable failure rates submitted by the panelists were 3.43% (minimum) and 10.1% (maximum), and the cut score was 60.6% (minimum) and 66.7% (maximum). As a result, a cut score of 63.8% (204 points) and the failure rate of 6.68% were derived (Fig. 2). The cut score, passing rate, and failure rate according to the yes/no Angoff method, the Hofstee method, and the traditional 60-point cut score are summarized in Table 4. Raw response data from panelists are available at Dataset 1.

## Discussion

### Key results

This study was conducted as a pilot test of converting the method of determining the cut score of the KMLE written exam from the current fixed pass rate of 60% of the total score. In this study, we developed the standard setting process based on the yes/no Angoff method, which could be applied throughout the development and implementation of the test items. The process was simulated with the data from the 86th KMLE written exam. As a result, the cut score was 198 in the yes/no Angoff method and 204 in the Hofstee method. Compared to the conventional 60% cut score, these are 6 points and 12 points higher, respectively. However, they can be considered more defensible cut scores, provided that those are derived by combining the multiple judgments made by individual specialists after a sufficient discussion of the minimum-competency physician.

### Interpretation

Based on our findings, it would be desirable to proceed with at least 3 rounds of determining the acceptance rate using the yes/no Angoff method. Modified Angoff methods have been widely used internationally in standard settings in various high-stake exams [11]. The panel’s opinion is presented in the first round based on the review and understanding of the items’ contents. In the second round, the panel has discussions. It comes to a consensus based on feedback, such as test takers’ score information, and the acceptance rate by the provisional acceptance line. This process should be carried out until a preset consensus is reached. It seems desirable to have panels refer to information on the difficulty and discrimination of the items after the third round, if possible.

The first and second cut scores obtained using the Hofstee method showed a difference of about 4 points (before starting standard setting discussion: 65.0% (208 points) and after the second round of the yes/no Angoff: 63.8% (204 points). Also, like the yes/no Angoff method, a lower cut score was derived during the second round than the first round. This result could not be identified in studies that conduct the Hofstee method only once for supplementary purposes. This finding suggests that, like the modified Angoff, an approach that allows panelists to change their judgment after discussion could also be useful for the Hofstee method. Further research will be needed on how many rounds would be required until the change in the cut score becomes minimal.

When comparing the 2 standard setting methods’ results in this study, the cut score obtained by the Hofstee method was lower than that obtained by the yes/no Angoff method. However, in a previous study, the Hofstee method derived a higher value by 5 to 6 points than the modified Angoff method [11]. Given that no gold standard yields a “perfect” passing score [10], neither can be considered a single “right value.” However, it can be argued that both cut scores obtained are generally within an acceptable range in that they are located between 61.9% and 67.8%, which is the range of cut scores derived from KMLE data from 2017 to 2019. Therefore, it would be more appropriate to consider that the Angoff method, which synthesizes the judgments of the independent test items, and the Hofstee method, which focuses on the whole test, have a complementary relationship. Above all, standard setting, which has undergone repeated discussions and agreements among panelists, can be considered in a more defensible way than a fixed 60% cut score, which has been conventionally used.

### Limitations

Since all the panelists who participated in this study were specialists in their fields, there was a limitation in that they had to make judgments on items outside their current practice areas. However, considering that the purpose of KMLE is to evaluate the competency of the basic medical education level, it can be assumed that all panelists have sufficient expertise to make judgments about the standard setting. Indeed, despite the initial variation, the differences between panels gradually narrowed during the discussion process. Since the process demanded as much as 800 minutes there is a feasibility limitation of used method. Therefore, further research will be needed to improve the efficiency of this process.

### Suggestions

The panel composition that participates in the cut score decision is essential. Not only do the panels perform simulations in advance, but they also need to be composed of primary care and medical education experts. While setting the standard using the modified Angoff method, the panel must understand the items’ contents as much as possible [12]. To this end, it is desirable to add commentary for each item while initially developing the test. Before entering the group discussion, the panel should be provided time to review the questions, and their opinions should be discussed afterward. This process will help the panel fully understand the content of the question. This process can be operated more efficiently by developing a computer system that immediately displays the discussion results and provides feedback to the panel. Finally, the transition to the new KMLE passing method requires supplementation of the legal system and a grace period of at least 2 years before implementation for prior notice to stakeholders.

### Conclusion

Based on 320 items of KMLE 2022, the yes/no Angoff and Hofstee methods derived cut scores of 198 points (failure rate=4.90%) and 204 points (failure rate=6.70%), respectively. Repeated discussions and the provision of opportunities for subsequent change in judgments have reduced the variation between panelists, not only in the yes/no Angoff but also in the Hofstee method. The final cut scores from both methods were similar to the findings of recent studies based on KMLE data, which suggest the acceptability of cut scores and the possibility of independent use of both methods.

## Figures and Tables

**Table 1.** Schedule of standard setting process

Time (min)	Process
40	Orientation for standard setting
40	Discussion for minimum-competency physician
	Break
210	Round 1 voting
	Break
150	Round 1 voting (continued)
90	Post-voting discussion (round 1)
60	Round 2 voting
40	Post-voting discussion (round 2)
30	Round 3 voting
20	Post-voting discussion (round 3)
20	Round 4 voting
20	Post-voting discussion (round 4)
20	Round 5 voting
10	Post-voting discussion (round 5, final round)
50	Wrap-up discussion
800 (total)	

**Table 2.** The composition of the panel

Specialty (subspecialty)	No. of panelists	Previous experience with KMLE
Internal medicine (gastrology)	1	
Internal medicine (cardiology)	1	Item development
Internal medicine (endocrinology)	1	Item development
Internal medicine (infectious disease)	1	
General surgery	1	Item review
Obstetrics and gynecology	1	
Pediatrics	1	
Psychiatry	1	Item review
Emergency medicine	1	
Family medicine	4	Item review
Preventive medicine	1	
Total	14	

KMLE, Korean Medical Licensing Examination.

**Table 3.** Cut score and passing rate of each round of standard setting

Round	Mean±standard error	Cut score	Passing rate (%)	Change of mean (compared with previous round)
1	217.43±4.45	217	88.20	NA
2	206.57±2.20	206	92.95	10.86
3	200.43±1.22	200	94.61	6.13
4	199.64±1.05	199	94.80	0.79
5	198.71±0.91	198	95.13	0.92

NA, not available.

**Table 4.** Comparison of cut score depending on standard setting methods

	Yes/no Angoff	Hofstee	60% correct (current cut score)
Cut score	198	204	192
Passing rate	95.1	93.3	96.6
Failure rate	4.90	6.70	3.40

## Footnotes

**Data availability**
Data files are available from Harvard Dataverse: https://doi.org/10.7910/DVN/SV6BVG Dataset 1. Raw data generated from yes/no Angoff and Hofstee methods for the standard setting of the Korean Medical Licensing Examination written test by panelists.

## Supplementary materials

Supplementary files are available from Harvard Dataverse: https://doi.org/10.7910/DVN/SV6BVG

Supplement 1. Audio recording of the abstract.
